# Supplementary material for: Adverse drug events leading to emergency department visits: A multicenter observational study in Korea
Source: PLoS One. 2022 Sep 19;17(9):e0272743. doi: 10.1371/journal.pone.0272743 (PMC9484687; doi:10.1371/journal.pone.0272743)
Supplement: S1 Table — ADE, adverse drug event; ED, emergency department. (DOCX) [file pone.0272743.s001.docx]

**S1 Table.** Causative drugs of ADE related ED visits according to age group

| **Total** | **Age group** | | |
| --- | --- | --- | --- |
|  | **Children/**  **adolescents**  **(n = 242)** | **Adults**  **(n = 1,088)** | **Elderlies**  **(n = 774)** |
| acetylsalicylic acid (10.4%) | influenza vaccines (8.3%) | acetylsalicylic acid (8.5%) | acetylsalicylic acid (12.5%) |
| warfarin (5.8%) | diphtheria-pertussis-poliomyelitis-tetanus (5.5%) | warfarin (3.8%) | warfarin (7.8%) |
| clopidogrel (3.3%) | pneumococcal vaccines (5.1%) | cefaclor (2.6%) | clopidogrel (5.3%) |
| influenza vaccines (3%) | Antibiotics (3.5%) | carboplatin (2.4%) | glimepiride (4.6%) |
| cisplatin (2.9%) | amoxicillin and enzyme inhibitor (3.5%) | cisplatin (2.3%) | cisplatin (3.7%) |
| glimepiride (2.5%) | Hemophilus influenzae B vaccines (3.1%) | influenza vaccines (2%) | metformin (3.6%) |
| metformin (2.2%) | dexibuprofen (2.8%) | fluorouracil (1.8%) | insulins (2.6%) |
| insulin (2%) | BCG vaccine (2.4%) | irinotecan (1.8%) | gemcitabine (1.9%) |
| cefaclor (1.9%) | meningococcal vaccines (2%) | antibiotics (1.7%) | tramadol (1.9%) |
| carboplatin (1.8%) | encephalitis, Japanese, live attenuated (2%) | cyclophosphamide (1.7%) | fluorouracil (1.6%) |
| tramadol (1.7%) | pneumococcus, purified polysaccharides antigen conjugated (2%) | doxorubicin (1.7%) | insulin glargine (1.6%) |
| fluorouracil (1.7%) | poliomyelitis vaccines (1.6%) | ibuprofen (1.7%) | amlodipine (1.4%) |
| gemcitabine (1.5%) | levodropropizine (1.6%) | paclitaxel (1.7%) | etoposide (1.4%) |
| doxorubicin (1.4%) | rota virus, live attenuated (1.6%) | tramadol (1.7%) | hydrochlorothiazide (1.4%) |
| cyclophosphamide (1.3%) | valproic acid (1.6%) | insulin (1.4%) | carboplatin (1.3%) |
| etoposide (1.3%) | vincristine (1.6%) | clopidogrel (1.4%) | valsartan (1.3%) |

ADE, adverse drug event
