# Supplementary material for: Adverse drug events leading to emergency department visits: A multicenter observational study in Korea
Source: PLoS One. 2022 Sep 19;17(9):e0272743. doi: 10.1371/journal.pone.0272743 (PMC9484687; doi:10.1371/journal.pone.0272743)
Supplement: S2 Table — ADE, adverse drug event; ED, emergency department. (DOCX) [file pone.0272743.s002.docx]

**S2 Table.** Causative drugs of preventable cases of ADE related ED visits (classified by the therapeutic main group)

| **Total** | **Age group** | | |
| --- | --- | --- | --- |
|  | **Children/**  **adolescents**  **(n = 18)** | **Adults**  **(n = 164)** | **Elderlies**  **(n = 145)** |
| Drugs used in diabetes (51.2%) | Anti-inflammatory and antirheumatic drugs (33.3%) | Drugs used in diabetes (36.7%) | Drugs used in diabetes (60.7%) |
| Antithrombotic agents (10.6%) | Antithrombotic agents (33.3%) | Antithrombotic agents (13.3%) | Antithrombotic agents (21.4%) |
| Psycholeptics drugs (8.8%) | Antidiarrheals, intestinal anti-inflammatory/anti-infective agents (33.3%) | Psycholeptics drugs (11.7%) | Psycholeptics drugs (24.2%) |
| Antiepileptics (3.5%) |  | Antiepileptics (8.3%) | Antihypertensive drugs (16%) |
| Analgesic drugs (2.4%) |  | Psychoanaleptics (5%) | Analgesic drugs (14.3%) |
| Thyroid therapy (2.4%) |  | Thyroid therapy (3.3%) | Thyroid therapy (11.1%) |
| Antihypertensive drugs (2.4%) |  | Stomatological preparations (3.3%) | Urological drugs (12.5%) |
| Psychoanaleptics (1.8%) |  | Ophthalmological drugs (1.7%) | Agents acting on the renin-angiotensin system (14.3%) |
| Agents acting on the renin-angiotensin system (1.8%) |  | Antihistamines for systemic use (1.7%) | Calcium channel blockers (16.7%) |
| Stomatological preparations (1.8%) |  | Cough and cold drugs (1.7%) | Beta blocking agents (20%) |
| Urological drugs (1.2%) |  | Anthelmintic drugs (1.7%) | Cardiac therapy (25%) |
| Calcium channel blockers (1.2%) |  | Analgesic drugs (1.7%) | Antiparkinson drugs (16.7%) |
| Beta blocking agents (1.2%) |  | Muscle relaxants (1.7%) | Antiepileptics (20%) |
| Cardiac therapy (1.2%) |  | Antineoplastic drugs (1.7%) | Anesthetic drugs (25%) |
|  |  | Agents acting on the renin-angiotensin system (1.7%) | Diuretic drugs (33.3%) |
|  |  | Antianemic drugs (1.7%) | Stomatological preparations (50%) |
|  |  | Antiobesity preparations, excluding diet products (1.7%) |  |
|  |  | Drugs for constipation (1.7%) |  |

ADE, adverse drug event; ED, emergency department
