# Supplementary material for: Adverse drug events leading to emergency department visits: A multicenter observational study in Korea
Source: PLoS One. 2022 Sep 19;17(9):e0272743. doi: 10.1371/journal.pone.0272743 (PMC9484687; doi:10.1371/journal.pone.0272743)
Supplement: Attachment — Submitted filename: PONE-D-21-39781_answer reviewer.docx [file pone.0272743.s003.docx]

**Reviewer 1:**

The study is very interesting for the pharmacovigilance area, as it makes a multicentric retrospective study to assess the frequency of adverse events in emergency admission. As mentioned by the author himself, what we have in the literature today with this objective are studies in a single health unit, thus limiting some data and comparisons. The article was well structured and shows interesting results, which can help in better preventive actions to avoid medication errors, which was the most important data of the article.

**Response:** Thank you for your positive comments and nice summary. We are submitting a revised manuscript that addresses the concerns raised. A detailed, point-by-point response to these concerns is attached.

I have some suggestions that may provide more support:

1) it would be interesting to demonstrate how many medications, on average, by age, the subjects took, and thus relate to the influence of polypharmacy on reactions and medication errors;

**Response:** We absolutely agree with your point of view in that polypharmacy and taking many medications may be associated with increased risk of adverse drug events (ADE). Unfortunately, since we enrolled subjects visiting to emergency department, it was not possible to analyze the medications or prescriptions by the other clinics or hospitals for each patient. Given that polypharmacy is frequently observed especially in older adults, it is speculated that polypharmacy might affect the development of ADE in older adults. We added this point as a limitation of this study (page 18, line299-304).

2) it would be interesting for medication errors to show a table of which medications are involved in these errors and the type of error, thus being able to direct public policies for the prevention of medication errors;

**Response:** Thank you for your comments. We agree with the reviewer’s comments that medication error is an important part to address in this real-world study on pharmacovigilance. However, since we did not analyse the causative drugs related with medication error, it is regrettable to present this data. Instead, we described and presented the medications related with preventable ADE in supplementary Table S2.

3) I believe that the major limitation of the study is that it was retrospective, and thus having to believe that everything was in the medical records, I suggest putting as a limitation - that the data presented may be greater, since the data were extracted from medical records, in hospitals different, different clinical care teams, and some reactions may not have been recorded, or even not identified, since some signs and symptoms are confused with diseases.

**Response:** Thank you for your helpful comments, which have substantially improved the quality of our manuscript. We also agree that the real body of ADE cases would be bigger than we estimated in this study. According to your suggestion, we described the limitation of this study in the discussion section (page 21, line 353-357).

**Review 2**

**1. Title**

I suggest to indicate the Country where the study was performed (i.e., a Korean multi-center…).
**Response:** Thank you for your comments. The title was modified including the country as “Adverse drug events leading to emergency department visits: A multicenter observational study in Korea”.

**2. Abstract**
1) I suggest to use visit instead of admission, seriousness instead of severity, adverse drug event (ADE) instead of side effect, serious instead of severe. Please modify these terms also in the manuscript and tables.

**Response:** We appreciate the reviewer’s helpful suggestion. We changed certain terms in the abstract and articles based on your suggestions. The word ‘admission’ was changed to the visits except for cases in which an actual ward is admitted. A total of eight ‘severe’ terms were replaced with ‘serious’. The term ‘severe’ was changed to ‘serious’ in statements conveying the severity of ADE in general. However, just as the CTCAE severity grading scale employs the term ‘severity’ rather than ‘seriousness’, the term severity and severity are interchangeable. The term ‘side effect' appears four times in the original manuscript. We used this term to refer to undesirable response due to pharmacologic property of drugs, such as antihistamine-induced sedation, as a part of type A reactions (overdose, secondary effects and drug interactions). Thus, we reserved the term ‘side effects’ for a certain type of ADE. To avoid confusion regarding this issue, we clarified the methods in detail.

2) Statistical analysis performed by the Authors should be added to the Abstract section (where is the multivariate logistic regression reported in the main manuscript?).

**Response:** Thank you for your comment. We added the statistical analysis in the abstract.

3) More detailed results should be added to the Abstract section (where are the interesting ORs reported in table 3?).

**Response:** Thank you for your helpful comments. We detailed the results of the abstract by adding OR and 95% CI in describing the main findings of the multivariate logistic regression analysis presented in Table 3.

*“Multivariate logistic regression analysis revealed that compared to non- adverse drug event-related cases, adverse drug event-related emergency department visitors were more likely to be female (60.6% vs. 53.6%, p<0.001, OR 1.285, 95% CI 1.025-1.603) and older (50.8 ± 24.6 years vs. 37.7 ± 24.4 years, p<0.001, OR 1.892, 95% CI: 1.397-2.297). Comorbidities such as diabetes, chronic kidney disease, chronic liver disease, and malignancies were also significantly associated with adverse drug event-related emergency department visits.”*

**3. Methods**
1) Which kind of classification system is used by the Author to describe both suspected (causative) medications and observed ADEs? I suggest to detail in this section both ATC and MedDRA classification systems.

**Response:** Thank you for pointing this out, which we did not fully acknowledge in our original manuscript. In this study, the World Health Organization Adverse Reaction Terminology (WHO-ART) was used to describe ADE instead of MedDRA. For the causative drugs, we used the Anatomical Therapeutic Chemical (ATC) Classification System. We modified the description of the methods used in the classification of ADE and causative drugs (page 8, line 124-129).

2) Which kind of algorithm/scale is used by the Author to estimate ADE preventability? There are several approaches, i.e. the Schumock and Thornton criteria, that could be described and used by the Authors.

**Response:** As you mentioned, we used and characterized the preventability of ADEs by the Schumock and Thornton criteria. the description for the criteria of preventability was added in lines 144-145 of the ‘main outcomes of ADEs’ section.

**4. Discussion**
This section should be more extensive and reader-friendly. Authors should perform a point-by-point (demographic, pharmacological and clinical characteristics) comparison between their results and those already published worldwide (i.e., Korea vs Europe, Korea vs United States, Korea vs Canada, Korea vs Australia, etc.). Some examples of similar analysis performed in Europe that could be useful in the aforementioned comparison are reported below: PMID: 32327995, PMID: 33708120, PMID: 34358104.

**Response:** We appreciate the reviewer’s helpful suggestion. We changed the discussion part in the order of incidence, elderly, and female. In addition, our study results were compared with other studies in the order of domestic and foreign studies. According to your comments, we further reviewed the pharmacoepidemiologic studies in Italy and described the summarisez results of the Italian study in the discussion section (page 19, line 320-323).

I also suggest to add here some points of strengths (or to better discuss the few points of strengths already mentioned). Which are the clinical implications in conducting this kind of pharmacoepidemiological studies? Why pharmacovigilance and pharmacoepidemiology approaches could be considered a valuable options in the evaluation of drug-safety in a real-life setting such as ED?

**Response:**  We absolutely agree with the reviewer’s comments that the pharmacovigilance and pharmacoepidemiology approaches offer great opportunity to suggest what is still needed to improve drug safety around the world. According to the reviewer’s suggestion, we added the strength and importance of the pharmacoepidemiologial study of ADE in real world in lines 319-333 and the conclusion.

**5. Figures**
The quality of figures is relatively low, please make them more readable.

**Response:** The low resolution of the pictures is due to PLOS one's e-submission system. We made the figures through R software with high resolution. Perhaps, when the paper is published, you will be able to see high-resolution pictures. Following this, we attached the PDF version of the high-resolution figures.
