# Supplementary material for: Adverse drug events leading to emergency department visits: A multicenter observational study in Korea
Source: PLoS One. 2022 Sep 19;17(9):e0272743. doi: 10.1371/journal.pone.0272743 (PMC9484687; doi:10.1371/journal.pone.0272743)
Supplement: Attachment — Submitted filename: Response to Reviewers.docx [file pone.0272743.s004.docx]

**Manuscript PLOS-D-21-39781R1**

**Response to reviewers**

Thank you for giving us the opportunity to submit a revised draft of the manuscript “adverse drug events leading to emergency department visits: A multicenter observational study in Korea” for publication in the PLOS One. We appreciate the time and effort that you and the reviewers dedicated to providing feedback on our manuscript and are grateful for the insightful comments on and valuable improvements to our paper. We have incorporated most of the suggestions made by the reviewers. Those changes are highlighted within the manuscript. Please see below, in red, for a point-by-point response to the reviewers’ comments and concerns. All page numbers refer to the revised manuscript file with tracked changes.

**Reviewers’ Comments to the Authors:**

Reviewer 2: Please revise the following sentence: “MEREAFaPS study group also reported that antiplatelet agents, anticoagulants, and antidiabetes were drugs such as antiplatelet agents and anticoagulants were a common cause of ADE-related ED visits in Italy”

Author response: Thank you for pointing this out. The reviewer is correct, and we revised the sentence following as “The MEREAFaPS study also reported that antiplatelet agents, anticoagulants and anti-diabetes were also common causes of ADE-related ED visits in Italy”
